# A Method for Quantitative Analysis of Standard and High-Throughput qPCR Expression Data Based on Input Sample Quantity

**DOI:** 10.1371/journal.pone.0103917

**Published:** 2014-08-04

**Authors:** Mateusz G. Adamski, Patryk Gumann, Alison E. Baird

**Affiliations:** 1 Neurology, SUNY Downstate Medical Center, Brooklyn, New York, United States of America; 2 Neurology, UJCM, Krakow, Poland; 3 Department of Physics, Harvard University, Cambridge, Massachusetts, United States of America; 4 Institute for Quantum Computing, Department of Physics and Astronomy, University of Waterloo, Waterloo, Ontario, Canada; Louisiana State University and A & M College, United States of America

## Abstract

Over the past decade rapid advances have occurred in the understanding of RNA expression and its regulation. Quantitative polymerase chain reactions (qPCR) have become the gold standard for quantifying gene expression. Microfluidic next generation, high throughput qPCR now permits the detection of transcript copy number in thousands of reactions simultaneously, dramatically increasing the sensitivity over standard qPCR. Here we present a gene expression analysis method applicable to both standard polymerase chain reactions (qPCR) and high throughput qPCR. This technique is adjusted to the input sample quantity (e.g., the number of cells) and is independent of control gene expression. It is efficiency-corrected and with the use of a universal reference sample (commercial complementary DNA (cDNA)) permits the normalization of results between different batches and between different instruments – regardless of potential differences in transcript amplification efficiency. Modifications of the input quantity method include (1) the achievement of absolute quantification and (2) a non-efficiency corrected analysis. When compared to other commonly used algorithms the input quantity method proved to be valid. This method is of particular value for clinical studies of whole blood and circulating leukocytes where cell counts are readily available.

## Introduction

Over the past decade a rapid increase has occurred in the understanding of RNA expression and its regulation. Quantitative polymerase chain reaction(s) (qPCR) have become the gold standard for measuring gene expression. Accurate analysis of qPCR data is crucial for optimal results and a number of well-defined methods are in use to calculate gene expression. These include the comparative C_T_ method [1], the efficiency corrected method [2] and sigmoidal curve fitting methods [3], all of which provide relative quantitative information. A standard curve of serial dilutions of a known sample is additionally required to measure the absolute number of transcript copies in a sample.

For most scientific purposes, relative quantification, expressed as fold change, is sufficient to provide the required information. Hence, the comparative C_T_ and efficiency corrected methods, as well as the sigmoidal curve fitting methods are widely employed, but each method has strengths and weaknesses. The comparative C_T_ method by Livak et al. [1] has the advantage of ease of use but is based on the assumption that transcript amplification efficiencies are 100%. In the efficiency corrected method by Pfaffl [2] the relative expression ratio is calculated only from the real-time PCR efficiencies and the crossing point deviation of an unknown sample versus a control. This model needs no calibration curve and gives improved quantification but is complex to use and requires determination of the amplification efficiency.

Furthermore, all of these methods require the use of reference (control or housekeeping) genes to correct for unequal amounts of biological material that may exist between the tested samples. The commonly used housekeeping genes were initially selected on the basis of their abundance and expression in a wide variety of tissues. An absolute requirement and widely held assumption of housekeeping genes has been that their expression is constant under all conditions and is unaffected by the experimental conditions [4]. However, the expression of commonly used housekeeping genes has since been found to vary considerably in many conditions [5]–[12]. In the case of *in vitro* or *ex-vivo* experiments it is usually possible to perform additional experiments to identify and validate appropriate control genes. In the case of clinical studies, however, where sample volumes are usually limited, it is rarely possible to test gene expression before and after the experiment (i.e., before and after the disease occurs).

The advent of next generation high throughput qPCR, based on reaction volumes scaled to the nanoliter range and with a consequent dramatic reduction in the volume of reagents and samples, has been a major advance for the analysis of clinical samples [13]. The Fluidigm Biomark system, one of the new high-throughput reverse transcription PCR (HT RT-qPCR) systems, permits up to 96 transcripts in 96 samples to be studied simultaneously during a single run, in a total of 9216 reactions. This allows many more transcripts to be studied from routine clinical samples, representing a 40 to 50 fold improvement in efficiency over standard qPCR [14], [15]. However, HT RT-qPCR has also raised new issues; for example, transcript amplification efficiency may be affected by potential interactions (i.e., primer dimer, competition) between multiple primers during the preamplification and amplification steps.

Here, we present a method for the measurement of the absolute gene expression for standard and high throughput qPCR experiments based on the input sample quantity. Based on this method three equations were developed: (1) for the measurement of fold change differences between target and control samples; (2) for the comparison of results from different experiments and different machines after normalization to a reference cDNA sample; (3) for analyses of samples of unknown efficiency. Gene expression results calculated using the input quantity method were then validated in a serial dilution series of commercial cDNA and using different starting cell concentrations. In clinical samples, fold change values calculated with the input quantity method were compared to values obtained using other commonly used algorithms. The input quantity method has the advantages of avoiding the use of control genes, of being efficiency corrected, and providing both fold change and absolute results. This method can also be applied in the verification and quantification of qualitative results from microarray studies for multiple genes.

## Theory

### 1. Requirements for the input quantity method

The input quantity method has several requirements. First, the amount of material used for RNA extraction has to be measured: for example, cell count is required for cell suspensions (e.g., peripheral blood mononuclear cells (PBMCs), lymphocytes and cell lines), white blood cell (WBC) counts are needed for whole blood studies and tissue volumes are needed for solid tissues. Secondly, for reverse transcription of RNA to cDNA the same reagents, volumes and protocols for a given experiment need to be used. Thirdly, the amplification efficiency and correlation coefficients (*R^2^*) should be assessed for each gene assay based on a standard dilution series. Finally, full application of this method requires the use of a standard sample (i.e., commercial cDNA – reverse transcribed cDNA from RNA extracted from all human tissues) for each measurement.

### 2. Mathematical model for qPCR amplification

As per Livak et al. [1], in the qPCR target cDNA sequence is amplified in an exponential fashion:
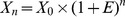
(1)where *X_n_* is the number of target cDNA molecules after n cycles, *X_0_* is the number of cDNA molecules before amplification, *E* is the efficiency of target cDNA amplification and *n* is the number of amplification cycles. In the case of perfect efficiency (*E* = 100%) the number of target cDNA molecules doubles every cycle.

In qPCR, the number of target cDNA molecules for a given sample is reflected by the threshold cycle - or according to the MIQE guidelines [4], quantification cycle (Cq) - because Cq is the intersection between an amplification curve and threshold. The threshold is the level of fluorescence above background fluorescence – set at the same level for all samples in the experiment. Each sample that crosses the threshold (regardless of the amplification cycle number) has the same fluorescence intensity hence the same target cDNA copy number.
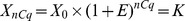
(2)where *XnCq* is the number of target cDNA molecules at the Cq, *nCq* is the cycle number at which amplification crosses the threshold and *K* is a constant value for all samples in a given experiment.

### 3. Analysis normalized to input sample quantity

In order to adjust the results of gene expression to unequal amounts of starting material the number of cells used for RNA extraction has to be incorporated into Equation 2.
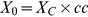
(3)where *Xc* is the transcript number per cell and *cc* is the number of cells used for RNA extraction (e.g., complete blood count for whole blood analysis, or hemocytometer cell count for cell subset analysis). Hence,



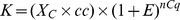
(4)Therefore to compare gene expression between target (T) and control (C) samples where *E* and *K* are the same for T and C, *ccT* is the input cell count for target sample and *ccC* is the cell input for the control sample. For the target samples the following formula is obtained:
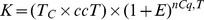
(5)where *T_C_* is the number of transcripts per cell in the target samples.

For the reference or control samples the following formula is obtained.
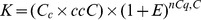
(6)where *C_C_* is the number of transcripts per cell in the reference samples.

As *K* is constant, Equations 4 and 5 equal each other:
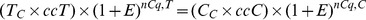
(7)


To obtain the comparison between target and control samples:
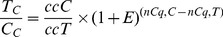
(8)


This way we can obtain the measure of gene expression expressed as a fold change difference between the test and control samples.

### 4. Analysis normalized to input quantity and normalized to standard cDNA

When a standard reference sample is introduced, for example a sample that contains a high concentration of studied transcripts, the following modifications are made, starting with Equation 2, *K* for sample *X* with a starting quantity of *cc* is:
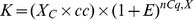
(9)


*K* for a standard cDNA of uniform quantity is:
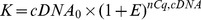
(10)


Normalizing to cDNA:
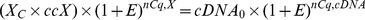
(11)

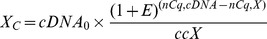
(12)


Since the number of transcripts before amplification in standard cDNA (cDNA_0_) is constant we may assume it is equal to 1 then:
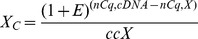
(13)


To obtain the comparison between test and control samples, the respective *T_c_* and *C_c_* are calculated using Equation 13. Then *T_c_* is divided by *C_c_* to obtain the measure of gene expression, expressed as a fold change.

### 5. Analysis normalized to input quantity and/or normalized to standard cDNA without known efficiency

If *E* for the working primers is not assessed in the experiment, one may make an assumption that the *E* equals 100% -then Equation 8 is:
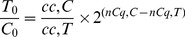
(14)


Whereas, adjusting to the standard cDNA sample, for sample X Equation 12 is:
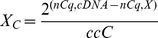
(15)


## Materials and Methods

To assess the reliability of the input quantity method, the stability of expression values calculated across serial dilutions of a standard cDNA sample and of different starting numbers of two samples of peripheral blood mononuclear cells (PBMCs) were determined. The validity of the input quantity method was assessed by comparison to fold changes obtained using the Livak [1] and Pfaffl [2] methods for three transcripts in a cohort of stroke patients and control subjects.

The Institutional Review Board at the State University of New York (SUNY) Downstate Medical Center approved the study. All study participants and/or authorized representatives gave full and signed informed consent. Where applicable, the conduct and reporting of the study are in accordance with the MIQE criteria [4]. The detailed laboratory protocols but not the data analysis described in this manuscript have been previously published [14].

### 1. RNA extraction and reverse transcription

Whole blood was obtained from 38 ischemic stroke patients between 7 and 90 days post stroke and from 17 sex- and race-matched control subjects. RNA was extracted using column separation (All-in-One Kit; Norgen Biotek, Thorold, Ontario, Canada) from 100 µl of whole blood and from a median of 2.0 million CD4^+^ cells. Peripheral blood mononuclear cells (PBMCs) from two control subjects were used for the cell dilution experiment, with RNA isolated from triplicate samples of 2 million, 1 million, 0.5 million and 0.25 million cells. Cellular counts (millions of cells per µl) were measured using a hemocytometer for CD4^+^ and for PBMCs; for whole blood, the total white blood cell count was obtained from the laboratory-measured complete blood count (CBC) in each study subject.

Density gradient centrifugation with Histopaque 1077 and 1119 (Sigma-Aldrich, St. Louis, MO) was used to separate the PBMC fraction from the whole blood. Positive magnetic bead separation (Miltenyi Biotec, Bergisch Gladbach, Germany) was used to separate CD4^+^ from PBMCs – the cellular purity was over 97%. The extracted RNA was resuspended in 50 µl of elution solution (All-in-One Kit protocol). cDNA was synthetized using the High Capacity cDNA Reverse Transcription Kit (Life Technologies, Carlsbad, CA), based on random hexamers, according to the manufacturer’s protocol. Following the protocol, the proportion of RNA solution to 2x RT master mix was 1∶1.

### 2. Primer development, RT qPCR and HT-RT qPCR

The primers for qPCR were self-designed, commercially synthesized by Invitrogen and wet tested using standard RT qPCR (StepOnePlus Real-Time PCR Systems; Applied Biosystems).

Standard RT qPCR (StepOnePlus Real-Time PCR Systems; Applied Biosystems) was used to measure the expression of *FDFT1* in the cell dilution experiment. Each sample and no template control were measured in triplicate. Based on a standard dilution series the efficiency for *FDFT1* in this experiment was 94%.

HT RT-qPCR was run on the BioMark HD System, using 96×96 Fluidigm Dynamic Arrays (Fluidigm, South San Francisco, CA). HT-RT qPCR was used first, to measure the expression of *FUT4*, *CD3E*, *FDFT1* and *B2M* in serial dilutions of commercial cDNA (Universal cDNA Reverse Transcribed by Random Hexamer: Human Normal Tissues; Biochain, Newark, CA) and second, to compare the expression of *FDFT1*, *CD3E* and *B2M* between control subjects and stroke patients in whole blood and CD4^+^ T lymphocytes. Two 5 point, four-fold serial dilution series of commercial cDNA were run in triplicate on two different plates. The volumes of commercial cDNA (diluent) in each dilution were: 100 µl (1∶1), 25 µl (1∶4), 6.25 µl (1∶16), 1.5625 µl (1∶64) and 0.39 µl (1∶256). According to the manufacturer’s protocol, the assay for each HT RT-qPCR experiment contained 10 µl of cDNA. The efficiencies for the genes, assessed with HT RT-qPCR, were: *B2M*- 87%, *FDFT1-* 86%, *FUT4-* 79% and *CD3E*- 79%. Five separate gene expression plates were used in this experiment. To normalize the gene expression results for stroke and control samples from different plates, a sample of commercial cDNA (containing high concentrations of all of the transcripts studied) of standard concentration and volume was run in duplicate on each plate. Each raw gene expression result (expressed as Cq) was normalized to the average Cq value for the same gene in the commercial cDNA samples that were run on the same plate (sample Cq value for gene X was subtracted from the average commercial cDNA Cq for gene X).

### 3. Calculation of fold changes

Fold change differences between stroke patients and control subjects for *B2M* and *CD3E* were calculated using the input sample quantity method according to Equation 13. The relative gene expression for *B2M* and *CD3E* were measured using the comparative C_T_ method of Livak et al. [1] and the efficiency corrected method of Pfaffl [2]. For these calculations *FDFT1* was used as control gene as its expression was not different in stroke patients compared to control subjects, based on the input quantity method (p>0.05).

### 4. Statistical analyses

The statistical analyses were performed using “R”, version 2.15.2. For the cDNA dilution analysis, linear regression modeling was used. For the cell dilution series, the data were analyzed using one way ANOVA, Welch’s correction for inhomogeneity of variances and post hoc *t*. tests with false discovery rate correction. For the analysis of the stroke versus control data, the 95% CI for the fold change values were calculated using the R package “mratios” and Dunnetts method; Wilcoxon rank sum tests were used for between group comparisons.

## Results

### 1. Gene expression measurements across different input volumes of a standard cDNA sample

**Figure 1:**
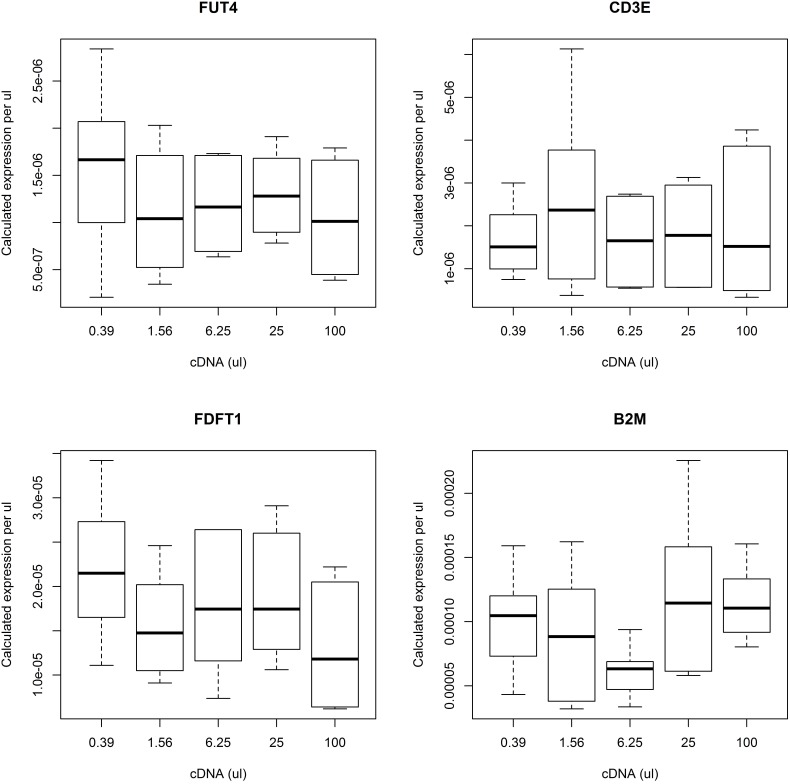
Expression of *FUT4*, *CD3E*, *FDFT1* and *B2M* in a standard dilution series of reference cDNA sample normalized to the volume of diluent using sample input quantity method.

To confirm the reliability of the sample input quantity method the expression of 4 transcripts (*FUT4*, *CD3E*, *FDFT1* and *B2M)* was measured in 5 point and 4-fold two serial dilutions of a standard cDNA sample. To measure the concentrations of each of the four transcripts in the standard cDNA sample, the results were normalized to the volume of diluent 100 µl (1∶1), 25 µl (1∶4), 6.25 µl (1∶16), 1.5625 µl (1∶64) and 0.39 µl (1∶256). Using this normalization procedure the same expression values were expected across the range of dilutions of the standard cDNA sample. The samples were run in triplicate on two separate plates giving 6 readings per input volume. The expression of all four genes calculated with the input quantity method was stable (Table 1, Figure 1). Detailed data are provided in a supplemental table (Table S1).

**Table 1:** Expression of *FUT4*, *CD3E*, *FDFT1* and *B2M* across serial volumes of a standard cDNA sample.

	*FUT4*	*CD3E*	*FDFT1*	*B2M*
**Coefficient**	–4.3e-8	–6.43e-8	–3.8e-7	–3.3e-6
**P value**	0.49	0.65	0.61	0.48
**R^2^**	–0.018	–0.028	–0.026	–0.016

Dilution coefficient, p and R^2^ values were obtained from linear regression analysis for each transcript.

### 2. Reliability of gene expression measurements across different starting numbers of cells

In order to determine the influence of variables present prior to the RT qPCR step (cell counting, RNA isolation and RT PCR) the expression of *FDFT1* in different starting numbers of PBMCs from two control subjects was measured. The raw data were normalized to the starting number of cells for each subject. The starting numbers of cells (2 million, 1 million, 0.5 million and 0.25 million) were within the range of the manufacturer’s recommendations for RNA extraction (All-in-One Kit, Norgen Biotec).

Based on the input quantity method the expression of *FDFT1* was significantly different across the input cell counts for both subjects (p = 1.4e-7, Subject 1 and p = 5.5e-5, Subject 2) (Table 2). Post hoc tests revealed that the expression of *FDFT1* in the 0.25 million input cell count in both subjects differed significantly from the other input cell concentrations: in Subject 1 (versus 2 million, p = 2.7e-6, versus 1 million, p = 0.00016 and versus 0.5 million, p = 7.6e-5) and in Subject 2 (versus 2 million, p = 5.9e-5, versus 1 million, p = 1.3e-6 and versus 0.5 million, p = 1.7e-6). Comparisons between the 2 million, 1 million and 0.5 million input cell numbers were not statistically significant for both subjects (p<0.05). Detailed data are provided in a supplemental table (Table S2).

**Table 2:** Expression of *FDFT1* in cell dilution series.

	2 million cells	1 million cells	0.5 million cells	0.25 million cells	p
**Subject 1**	0.26±0.01	0.23±0.02	0.24±0.06	0.15±0.02**	<<0.01
**Subject 2**	0.049±0.003	0.041±0.005	0.043±0.015	0.072±0.013**	<<0.01

p values were calculated using a one-way ANOVA.

**Post hoc tests revealed that expression of *FDFT1* in the 0.25 million input cell count differed significantly from the other input cell concentrations in both subjects.

### 3. Expression of CD3E and B2M in the late phase of stroke and in control subjects calculated using three methods

To assess the validity of the input quantity method using clinical samples, the expression of *CD3E* and *B2M* in whole blood and in CD4^+^ T lymphocytes was compared between patients in the delayed phase of stroke and control subjects. Fold change differences in gene expression were measured using the input quantity method (normalized to cell count), and the Livak and Pfaffl methods.

By all methods *B2M* expression was significantly increased in whole blood in the delayed phase of stroke and *CD3E* was significantly increased in CD4 cells (Table 3). No alterations in the expression of *CD3E* were found in whole blood. A borderline increased in *B2M* expression in CD4 cells was found using the input quantity method. Detailed data are provided in a supplemental table (Table S3).

**Table 3:** Fold change difference in the expression of *B2M* and *CD3E* in late phase stroke versus control subjects.

	*B2M*	*B2M*	*B2M*	*CD3E*	*CD3E*	*CD3E*
Wholeblood	Input QuantityMethod	Livak	Pfaffl	Input QuantityMethod	Livak	Pfaffl
Fold Change	2.51	2.19	2.28	1.27	1.12	1.22
95% CI	1.26, 15.89	1.26, 5.94	1.32, 6.20	0.67, 3.34	0.70, 2.01	0.79, 2.07
p	0.017	0.006	0.003	0.19	0.48	0.42
**CD4**						
Fold Change	1.35	0.57	0.70	3.13	1.78	2.10
95% CI	0.94, 2.17	0.26, 1.15	0.41, 1.23	1.61, 25.8	1.16, 3.42	1.35, 4.25
p	0.02	0.4999	0.26	2.10e-05	0.0084	7.50e-05

Fold change and 95% CI calculated using the 3 methods; data analyzed using R package “mratios” and Dunnetts method; Wilcoxon rank sum tests used for between group comparisons.

## Discussion

Several gene expression analysis methods are in common use, but the input quantity approach presented here offers two major advantages. Firstly, this method is independent of control genes. Secondly, with the assumptions of 1) uniform efficiency of RNA extraction and RT qPCR and 2) a constant concentration and volume of a standard sample, this method permits absolute quantification, expressed as the fraction of transcripts in the standard sample, across different experiments. The proposed algorithm is efficiency corrected, although analysis of results without known efficiency is also possible. With the use of a standard sample, the input quantity method also permits the comparison and analysis of results from different batches and results acquired on different qPCR machines. Furthermore, with the advent of HT RT-qPCR, this analytical method is also very useful for clinical research, where sample volumes are limited.

Our analyses show that the sample input quantity method permits gene expression to be measured across a wide range of commercial cDNA. Although the performance of both RNA extraction and RT qPCR may differ significantly across different cell concentrations and kits [15], our results show that, using the same protocol and reagents within the input quantities we tested, these variables can be successfully controlled. Furthermore, the expression of *B2M* and *CD3E* in study subjects calculated using three methods was highly concordant.

The rationale for the use of housekeeping (or control or reference genes) is to correct gene expression results, reflected as differences in Cq values between target and control samples, that could result from two main factors: different amounts of starting material or different levels of expression. Traditionally, housekeeping genes have been chosen on the basis of their abundance, ubiquitous expression across tissues and the assumption that their expression is stable under physiological and experimental conditions. However, the expression of conventionally used housekeeping genes varies considerably in many conditions. Therefore, reference gene selection requires additional experiments to validate gene expression stability under different experimental conditions [6]–[12], [14]. In many conditions, especially in the clinical setting, it is not possible to measure the effect of the disease/condition on reference gene expression.

The algorithm used for our sample input quantity method employs normalization to the sample input quantity (cell count, tissue volume etc.), which in result permits an absolute gene expression analysis. This method varies from the relative analysis approach, where results are normalized to reference gene expression. Due to normalization to the input quantity (measured in absolute scale) the measure of gene expression remains absolute, as in our method. In contrast, the gene expression from the relative analysis approach is based on the normalization to reference gene expression. Thus the ratio of the target gene expression to the reference gene expression represents a relative measure. By introducing a standard sample (of a stable transcript concentration), our method allows us to compare gene expression between different experiments. Instead of directly measuring transcript copy number- as it is commonly done in absolute measurements of gene expression- in our method, the measured gene expression is presented as a fraction of transcripts present in the standard sample. This fraction can be converted to the transcript copy number by measuring concentration of the target gene in the standard sample.

The input quantity approach presented here can be applied to clinical studies, to verify and quantitate microarray results, and to large scale studies of gene or microRNA expression. Having knowledge of the input cell count for all samples and the use of a uniform standard, first, allows normalization to the amount of starting material, and second, the use of the same standard allows normalization of results between different laboratories and different equipment.

## Supporting Information

Table S1 **Detailed data for analysis in Result Section 1.**
(XLSX)

Table S2 **Detailed data for analysis in Result Section 2.**
(XLSX)

Table S3 **Detailed data for analysis in Result Section 3.**
(XLSX)

## References

[1] Livak KJ, Schmittgen TD (2001) Analysis of relative gene expression data using real-time quantitative PCR and the 2(-Delta Delta C(T)) Method. Methods 25: 402–408. 11846609 10.1006/meth.2001.1262

[2] Pfaffl MW (2001) A new mathematical model for relative quantification in real-time RT-PCR. Nucleic Acids Res 29: e45. 11328886 10.1093/nar/29.9.e45 PMC55695

[3] Liu M, Udhe-Stone C, Goudar CT (2011) Progress curve analysis of qRT-PCR reactions using the logistic growth equation. Biotechnol Prog 27: 1407–1414. 21766473 10.1002/btpr.666

[4] Bustin S a, Benes V, Garson J a, Hellemans J, Huggett J, et al (2009) The MIQE guidelines: minimum information for publication of quantitative real-time PCR experiments. Clin Chem 55: 611–622. 19246619 10.1373/clinchem.2008.112797

[5] Dheda K, Huggett JF, Bustin S a, Johnson M a, Rook G, et al (2004) Validation of housekeeping genes for normalizing RNA expression in real-time PCR. Biotechniques 37: 112–119. 15283208 10.2144/04371RR03

[6] Vandesompele J, De Preter K, Pattyn F, Poppe B, Van Roy N, et al (2002) Accurate normalization of real-time quantitative RT-PCR data by geometric averaging of multiple internal control genes. Genome Biol 3: RESEARCH0034. 12184808 10.1186/gb-2002-3-7-research0034 PMC126239

[7] Chang TJ, Juan CC, Yin PH, Chi CW, Tsay HJ (1998) Up-regulation of beta-actin, cyclophilin and GAPDH in N1S1 rat hepatoma. Oncol Rep 5: 469–471. 9468581 10.3892/or.5.2.469

[8] Feroze-Merzoug F, Berquin IM, Dey J, Chen YQ (2002) Peptidylprolyl isomerase A (PPIA) as a preferred internal control over GAPDH and beta-actin in quantitative RNA analyses. Biotechniques 32: 776–782. 11962599 10.2144/02324st03

[9] Nishimura M, Nikawa T, Kawano Y, Nakayama M, Ikeda M (2008) Effects of dimethyl sulfoxide and dexamethasone on mRNA expression of housekeeping genes in cultures of C2C12 myotubes. Biochem Biophys Res Commun 367: 603–608. 18191039 10.1016/j.bbrc.2008.01.006

[10] Lin J, Redies C (2012) Histological evidence: housekeeping genes beta-actin and GAPDH are of limited value for normalization of gene expression. Dev Genes Evol 222: 369–376. 23099774 10.1007/s00427-012-0420-x

[11] Sikand K, Singh J, Ebron JS, Shukla GC (2012) Housekeeping gene selection advisory: glyceraldehyde-3-phosphate dehydrogenase (GAPDH) and β-actin are targets of miR-644a. PLoS One 7: e47510. 23091630 10.1371/journal.pone.0047510 PMC3472982

[12] Li R, Shen Y (2013) An old method facing a new challenge: re-visiting housekeeping proteins as internal reference control for neuroscience research. Life Sci 92: 747–751. 23454168 10.1016/j.lfs.2013.02.014 PMC3614345

[13] Devonshire AS, Sanders R, Wilkes TM, Taylor MS, Foy C a, et al (2013) Application of next generation qPCR and sequencing platforms to mRNA biomarker analysis. Methods 59: 89–100. 22841564 10.1016/j.ymeth.2012.07.021

[14] Adamski MG, Li Y, Wagner E, Yu H, Seales-Bailey C, et al (2013) Next-generation qPCR for the high-throughput measurement of gene expression in multiple leukocyte subsets. J Biomol Screen 18: 1008–1017. 23690294 10.1177/1087057113489882

[15] Spurgeon SL, Jones RC, Ramakrishnan R (2008) High throughput gene expression measurement with real time PCR in a microfluidic dynamic array. PLoS One 3: e1662. 18301740 10.1371/journal.pone.0001662 PMC2244704

